# Challenges of care coordination for complex patients among family medicine residents in a community ambulatory clinic: a qualitative study

**DOI:** 10.1186/s12909-024-05543-7

**Published:** 2024-05-23

**Authors:** Moses Tan Mong Heng, Gilbert Yeo Tian Seng, Eng Sing Lee

**Affiliations:** 1 National Healthcare Group Polyclinics, 21 Geylang East Central, Singapore, 389707 Singapore; 2 Department of Transitional Care, Woodlands Health, 17 Woodlands Dr 17, Singapore, 737628 Singapore; 3 National Healthcare Group Polyclinics, 3 Fusionopolis Link, Nexus @ one-north, Singapore, 138543 Singapore; 4 Lee Kong Chian School of Medicine, Singapore, 11 Mandalay Road, 308207 Singapore

**Keywords:** Primary care, Residency, Singapore, Qualitative interviews, Care coordination, Complex patients

## Abstract

**Background:**

Care coordination has been identified as one of five focuses of HealthierSG. Family medicine residents are expected to collaborate with other healthcare professionals for complex patients by the end of residency. However, many residents felt that it was challenging to coordinate care effectively among healthcare stakeholders. However, to date, no qualitative studies have explored these challenges. Therefore, this study aimed to understand the challenges encountered by family medicine residents when coordinating care for complex patients.

**Methods:**

This was a qualitative descriptive study in which semi structured in-depth interviews were conducted and guided by a topic guide. Total population sampling of 15 third-year family medicine residents in the National Healthcare Group Polyclinics was performed. The interviews were performed over Zoom and were transcribed. Thematic analysis was subsequently performed to analyse the transcripts. Coding was performed iteratively by two independent researchers. Disagreements were adjudicated by a third coder. A coding framework was agreed upon. Potential themes were then independently developed based on the coding framework.

**Results:**

Six themes emerged from the data, namely, interprofessional communications, accessibility, personal knowledge, time constraints, patient factors and caregiver dissent.

**Conclusion:**

Challenges faced by family medicine residents are multifaceted. While a few are systemic and pertain to the broader healthcare framework, others, such as issues of unfamiliarity with institutional workflows, community resources, and confidentiality, pertain to the microcosm of residency itself. These are reversible areas for improvement. These challenges can be addressed during planning of residency curricula to better equip family medicine residents with coordinating care for complex patients in the future.

## Background

The Agency for Integrated Care of Singapore defines complex patients as those fulfilling at least two of the following three health domains: complex medical issues (three or more chronic conditions or advanced disease), functional impairment (requiring assistance in one or more activities of daily living or cognitive impairment) or psychosocial impairment (caregiver issues, family issues, financial issues, social isolation or psychological issues) [1]. As Singapore experienced rapid aging, the prevalence of chronic conditions has increased. Interaction between chronic conditions adds to the complexity of management. In addition, the complexity of chronic disease, coupled with psychosocial issues, reduces individuals’ ability to seek help and self-manage care while simultaneously rendering care delivery more complex [2, 3].

Among the many strategies available for enhancing care delivery for complex patients, care coordination has been identified by the World Health Organisation (WHO) as a global priority for reorienting health services to the needs of complex patients [4]. Care coordination has become increasingly necessary as patients become more chronically ill, and our healthcare system has become more complex and fragmented [5]. When performed correctly, care coordination reduces hospital admissions; improves the quality of chronic disease management, patient satisfaction, and provides better access to specialty care [6]. The need for greater collaboration and coordination of care has also gained greater recognition in Singapore as mobilising community partnerships and constructing an integrated health and social ecosystem have been identified as key focuses of the HealthierSG initiative. Healthier SG is a multi-year initiative by Singapore’s Ministry of Health to shift the focus from reactively caring for those who are sick, to proactively preventing individuals from falling sick. This requires a systems effort to integrate care and support community partnerships, as well as a paradigm shift for the population to reshape their behaviors and lifestyles [7].

Despite the growing emphasis on care coordination, current research performed among family physicians in Singapore has revealed key areas of deficiency, such as inadequate knowledge of care prioritisation, patient characteristics such as cognitive or memory problems, poor social support, and low levels of motivation, hampering efficient care coordination between healthcare professionals for complex patients [8]. To date, no similar studies have been conducted on family medicine residents, who are expected to be proficient in this aspect after the completion of a three-year family medicine residency programme [9]. The ability to standardise the teaching of care coordination is also made even more challenging due to marked heterogeneity in the residency curriculum between institutions.

In National Healthcare Group Polyclinics (NHGP), family medicine residents are exposed to the breadth of family medicine through chronic disease care in the polyclinic interspersed with rotations to hospital outpatient clinics. At the end of residency, the Accreditation Council for Graduate Medical Education (ACGME) requires residents to perform effective coordination of patient-centered care during patient consultations with nurses, allied health, across different disciplines and specialties and to advocate for safe and effective transitions of care within and across healthcare delivery systems, including outpatient settings [10].

Effective care coordination can be understood from the conceptual framework laid out by the WHO which categorises care coordination into three major aspects, namely, sequential coordination, parallel coordination and indirect influences on provider relations [4, 11]. Sequential care coordination entails coordinating cross-sectoral transfers of care between tertiary and primary care. Parallel coordination of care encompasses co-working within multidisciplinary healthcare teams located in a common setting. Indirect influences on provider relations refer to any intervention to encourage both internal and external coordination [11].

We aim to uncover the additional challenges, both known and unknown, that residents face in aspects of care coordination. Understanding these challenges would allow educators to facilitate this domain during residency training, so that they could be better prepared to coordinate care for complex patients when they become family physicians.

## Methods

### Study participants

Total population sampling was carried out to recruit all 15 third-year family medicine residents from NHGP who had experience with the care of at least three complex patients in the area of care coordination. We excluded first- and second-year family medicine residents as the majority of their time are spent at providing care in the hospital setting.

Eligible participants were identified by a name list provided by the residency administrators and consent was sought by residency administrators for the research team to contact the participants. Subsequently, we contacted all third-year family medicine residents in NHGP by telephone, provided information on the research, confirmed their eligibility and scheduled a timing for consent taking. Participation in the research was voluntary. The study team was not involved in any direct supervisory role or part of the core teaching faculty to ensure that participation was voluntary. Adequate time was given for participants to consider their decision to participate in the study. The interviews were then conducted on separate occasions after consent was obtained. Ethical approval was obtained from the National Healthcare Group Domain Specific Review Board (Ref: 2020/01377).

### Setting

NHGP forms the primary healthcare arm of the National Healthcare Group (NHG). It operates eight polyclinics that serve a significant proportion of the population in the central and northern parts of Singapore. NHGP provides a comprehensive range of health services for the family, including treatment for acute medical conditions, management of chronic diseases, women & child health services, and dental care. The focus of NHGP’s care is on health promotion and disease prevention, early and accurate diagnosis, and disease management through physician-led team-based care.

NHG family medicine residents run resident care continuity (RCC) clinics once a week at their base polyclinics. There are 15 third-year family medicine residents. Some of these RCC clinics are embedded within a team consisting of a care manager and a care coordinator. In addition to the health services described above, family medicine residents also review patients discharged from inpatient settings and specialist outpatient clinics. Family medicine residents can then take over their care and continue community follow-up longitudinally.

### Data collection

Data were collected from January 2021 to January 2022 through individual in-depth interviews performed via video conferencing over Zoom® in view of pandemic restrictions [12].

Socioeconomic demographic information, including age, sex, ethnicity as well as school of undergraduate medical degree, post graduate postings and number of post graduate medical degrees were sought before the start of the interview. This was followed by a semi structured interview conducted by the principal investigator.

An interview guide was designed with reference to the conceptual framework laid out by the WHO on effective care coordination, existing literature and discussion among the research team members. A sample of the main interview questions is provided in Table 1. The interview guide consisted of questions on participants’ background and understanding of care coordination, followed by questions about the perceived challenges of care coordination for complex patients. The interviews proceeded in a way consistent with natural conversation and questions were not necessarily covered in the stated order. The participants were given the opportunity to discuss freely based on the questions asked. Probes and follow-up questions were used throughout the interview to clarify and to facilitate discussion. The interview questions were narrowed and modified over the course of the study using an iterative process based on the content of previous interviews.

**Table 1:** Interview guide

Interview questions
What was your background before entering residency?
What do you understand about complex patients?
What do you understand about care coordination?
How was your practice structured?
Which groups of healthcare professionals have you worked with in coordinating care with complex patients?
Could you share with us your experience at coordinating care for complex patients?
What are these challenges that prevent you from effectively coordinating care for complex patients? *Interviewers might ask questions pertaining to issues with sequential coordination, parallel coordination or indirect influences affecting care coordination.*
What are the most important points that you would like to highlight regarding care coordination for complex patients?

The interviews were digitally audio-recorded and transcribed verbatim. The transcripts were then verified by the principal investigator via audio recordings. Relevant fieldnotes, observations and reflexive diaries were also recorded during and after the interviews.

### Data analysis

A qualitative descriptive research methodology was chosen to explore the perspectives on challenges that residents face with regard to care coordination for complex patients. This methodology allows investigators to remain close to the data to obtain an accurate and detailed representation of the residents’ experiences and perceptions [13].

Using qualitative thematic analysis [14], the research team (MTMH, GYTS, LES) coded a sample of transcripts independently, before meeting to discuss and agree on a coding framework that was then applied to all transcripts. The qualitative findings are reported using the Consolidated Criteria for Reporting Qualitative Research (COREC) [15].

## Results

### Participant characteristics

All 15 third-year residents from NHGP provided consent for the interviews. There were 11 female participants. The mean age of the 15 participants was 30 years (range from 29 to 32 years). Most participants obtained their undergraduate degree from Yong Loo Lin School of Medicine, Singapore, with two who obtained their degree from the University of Malaya and one from King’s College London. Two participants had postgraduate diplomas in Family Medicine (GDFM) [16]. Six participants had prior experience working in a polyclinic setting before residency, while three participants did not have any prior experience working in other postings as medical officers before residency.

Six overarching challenges faced by residents in coordinating care for complex patients in primary care were shared.

### Challenges with interprofessional communications

#### Paucity of handovers

Residents reported that handover notes between healthcare professionals might be absent or that they may not contain important details on areas to follow up on. This results in the omission of essential information, which affects subsequent consultations.

Sometimes when I look through my colleague’s handover notes, I do not find anything about discussion; you know, referring to specialists and all. Therefore that part I feel is very challenging because, I do not know what to tell the patient. (Resident 3)

We may discuss or have the patients share things that are more than what we handover, because we only handover a summary, rather than the details. Therefore, important details might sometimes be lost along the way. (Resident 4)

### Overreliance on physical memos

Another reason behind poor interprofessional communications stems from overreliance on physical memos. Sometimes, patients forget to bring these memos for their appointments. Residents also lamented that memos that are too long would lead to information fatigue, while those that are too short lack clarity on areas for primary care to follow up on.

 In addition, there are scenarios in which the patient forgets to bring the memo. (Resident 1)

 When we see post discharge complex patients, we get a memo that is like a copy of the discharge summary; it is not very specific. Therefore, we must take time to read through and decipher the issues that need to be addressed in the polyclinic. (Resident 3)

 However, sometimes, certain unresolved matters in the hospital are not mentioned in the memo, so we are also not sure whether we should follow up on those issues. (Resident 11)

### Challenges with accessibility to care

#### Absence of a common platform for accessing information

Residents highlighted that the absence of a common platform across the nation has affected their ability to access patients’ clinical progress notes from other institutions. This results in an overreliance on the patient to provide subjective information on their progress, which is often challenging in view of heterogeneity in patient health literacy.

 We can see some hospitals’ clinic notes, but we do not have access to other hospitals’ clinic notes as they do not function on the same platform as us. Therefore, it is quite challenging to truly know why the patient is here and exactly what you need to do, especially when the patient himself also has no idea what needs to be reviewed. (Resident 2)

 Despite having NEHR, we are still unable to see the clinical records of some hospitals, especially for their outpatient clinics. (Resident 4)

### Conflicts arising from patient confidentiality

Confidentiality was mentioned by the participants as a stumbling block to care coordination in complex patients. Residents are uncertain regarding the limits of case discussion for fear of infringing the Personal Data Protection Act (PDPA) [17]. Because of the perceived issues surrounding the PDPA, they are unsure of what could or could not be shared.

 For referrals to the Emergency Department, I will check the NEHR on the same day to ensure that the patient has gone. However, for referrals to outpatient clinics, I cannot check the NEHR more than one day after the consult due to PDPA. Hence, I would not know whether the patient has gone. (Resident 13)

 We are not supposed to discuss with other residents or other doctors, who are not involved in the patients’ care because of PDPA. The problem lies if it is truly a complicated case, I would not know what can or cannot be shared; and whether I would be infringing on PDPA. (Resident 14)

### Inaccessibility to partners

Care coordination is a challenge because specialists and community partners are not as accessible or responsive as participants would like them to be at this moment in time, when residents would like to discuss the care of complex patients with them. Logistical barriers and lack of responses from partners are some of the reasons cited by participants.

 If you have proper contact for certain complex issues or conditions, then I think it will be great. However, I do not have the channel to contact these specialists. (Resident 11)

 It is quite difficult to call a neurologist or even find a number because we do not have access to the directories of hospital doctors. (Resident 6)

 I tried calling or emailing the specialists, but I did not receive any reply. (Resident 8)

### Challenges with knowledge deficiencies

Residents also cited personal knowledge deficiencies in identifying suitable community resources for organising care. They also highlighted difficulties keeping pace with rapidly evolving institutional workflows.

 I am not sure of the community partners whom we can tap on to coordinate care, such as social services, and how we go about getting these. (Resident 11)

 My lack of knowledge stems more from the workflows of the institution to understand its process and how to seek help for these complex patients who need different clinical services to coordinate their care. Nobody taught me all this. (Resident 12)

### Challenges with time constraints

Due to the lack of time in the polyclinic, participants feel challenged that they would usually have to prioritise the management of medical issues over social issues due to the lack of time. They would have to defer these social issues to the next appointment. Residents are also concerned that these important social issues may also not be addressed in the next appointment as the patient might be too medically complex. Residents lamented that because of a lack of time, it is difficult to perform anything additional for the patient, such as coordinating care with community partners for better health outcomes.

 With the pressure of time, we would want to prioritise medical issues over social issues. Sometimes, we are also not sure whether these social issues will be addressed in the next appointment because the patient is just too complex. (Resident 5)

 Because we are always very rushed, it is very difficult to do anything extra for the patient such as coordinating care with other parties, other than a typical consultation of history, physical examination and medical management. (Resident 1)

### Challenges with patients

#### Conflicts with respecting patient autonomy

Another major impediment to care coordination for complex patients is the patients themselves and their unwillingness to receive care. Residents sometimes feel that they are left with no choice but to respect the patients’ autonomy at the expense of optimal management for their complex medical conditions.

 Sometimes we do have such patients where they have very fixed beliefs, and when you try to challenge them, they are also not keen to listen. (Resident 7)

 If failing all my advice, he is still not keen; I will have to respect his autonomy to decide against the referral, which might to his health detriment. (Resident 9)

### Poor health literacy

Residents also described that a patient’s poor health literacy can greatly impact his or her ability to coordinate care. As complex patients have many medical conditions, they must have clear understanding of their medications and clinical management. Poor knowledge of their medical conditions can lead to a high rate of appointment defaults and affect residents’ ability to plan shared care with other members of the healthcare team, as patients do not turn up for their appointments in the first place.

 I think the classic examples will be those elderly with many conditions, yet they are unaware of their medical conditions. Sometimes, you know that if you let them out the door, they would still be lost, and they would continue to default all their subsequent appointments with the specialists or allied health. (Resident 8)

 Patient health literacy is an important obstacle to coordinating care. (Resident 9)

### Challenges with caregivers

#### Caregivers with differing priorities

Participants cited caregivers with differing priorities as the most widely encountered and recurring reason that caregivers gave against better coordination with other healthcare professionals. They cited work exigencies and lack of suitable time to bring their relatives to polyclinics for consultations with other healthcare professionals.

 The most common thing is that they tell us that they are struggling with finances, but when you offer a referral to the medical social worker, they declined because they find that it is too inconvenient to make a separate trip down to the polyclinic to see him. (Resident 13)

 This is not because the patient does not need resources; rather, the problem is because the caregiver thinks that it is very troublesome. (Resident 14)

#### Multiple caregivers

Having multiple caregivers and spokespersons attending to the care of the patient would also impair care coordination between caregivers, patients and other healthcare professionals. Residents spoke about different caregivers having varying views on the care of their loved ones, and sometimes these views might conflict, leading to confusion between patients and healthcare teams.

 If there are too many caregivers on board, this might pose an issue, especially if the patient is quite complex and a decision has to be made whether to engage the specialists or refer the patient to community resources. This prolongs the consultation. (Resident 9)

 The family is also slightly lost because different children, who are in charge of different areas of the patient’s care, still have different opinions on his care. (Resident 5)

## Discussion

In this study, the participants provided insights into the challenges they faced as family medicine residents towards care coordination for patients with complex needs. These challenges stem from many factors, including interprofessional communication, accessibility, personal knowledge, time constraints, patient factors and caregiver dissent. Nonetheless, all participants remained committed to the concept of collaboration between themselves and other healthcare professionalisms, despite these challenges. Residents also comprehend that their practice of medicine cannot be performed in silos. Coordination and collaboration with community and tertiary partners are necessary to ensure that complex patients do not fall through the cracks, but are well supported at every level of healthcare touchpoint.

However, the implementation and administration of care coordination with partners leave much to be desired. Family medicine residents highlighted important challenges associated with sequential coordination, such as inaccessibility to specialists and overreliance on physical memos for communication, with the unfortunate part of patients serving as deliverymen to deliver letters from one doctor to the nextOn the whole, communications with specialists seemed to be either one-way or delayed. Due to the paucity of cross-talk and exchange of ideas to develop a shared care plan for complex patients, family medicine residents feel isolated and unsupported. Care fragmentation has implications for both their learning about care collaboration and patient safety. Therefore, much can be done to align the paradigms of specialists in tertiary hospitals with those of primary care providers in the overall management of complex patients.

Within the institution, inadequate handovers between healthcare professionals and time constraints are some of the parallel challenges highlighted by residents that impede their efficiency at care coordination. While time constraints are a systemic concern, ensuring proper handovers within the institution can be inculcated to improve communication and collaboration.

As learners, the lack of knowledge on workflows and community resources are important indirect influences on internal and external coordination between healthcare professionals. While community resources are taught to residents, these are taught in a bolus manner. Perhaps, one could consider adopting spaced repetitions to encourage retention, as spacing out encounters with materials over time produce superior long-term learning, compared with repetitions that are massed together [18]. As such encounters are also more often caught than taught, educators could also use opportunities during patient encounters to teach residents how care could be collaborated with the community.

Although there is a paucity of studies focusing on the challenges faced by family medicine residents in coordinating care for patients with complex needs, several studies have been performed within primary care settings; these findings resonate with our findings. Locally, a study examining the public primary care system using a public care model identified the overreliance of physical memos, absence of a common platform and inaccessibility of specialists to respond in a timely manner as the main challenges faced at care coordination [19]. Another study also described ineffective communication and handovers between staff, resulting in a lack of care coordination and threats to patient safety [20]. The ineffective communication between primary and secondary care was also supported by another study that found delayed replies in response letters and omission of important details such as current medications and changes to medications lists. This might further impede patient safety [21]. Finally, a systematic review of qualitative studies on family physicians’ care for complex patients highlighted issues related to care prioritisation and patient characteristics, such as cognitive or memory problems, poor social support, and low levels of motivation hampering care coordination for complex patients [22]. Even though participants in the cited studies were primary care physicians and not residents, the corroboration of themes from other studies with our study reflects its pervasiveness among doctors. These are therefore important areas to look into to enhance care coordination and transitions in our healthcare ecosystem.

Despite these similarities, this study contributes new perspectives on the challenges of residents face in care coordination in areas of self-recognition of workflow unfamiliarity and the double edged sword of confidentiality. Some of the findings might differ from those of studies involving family physicians. First, there is a lack of knowledge on workflows. While residents are concerned about keeping up with changing workflows, family doctors from other countries are worried about the feasibility of applying these guidelines in a patient-centered manner, instead of a disease-specific manner [8]. This difference between these two groups may be attributed to the lack of constant presence in the primary care setting during the early years of residency. The inherent concerns regarding workflows may also be related to their inability to regard patients as a whole and to prioritise their issues. Keeping abreast of workflows might be a coping mechanism to ensure that patients are at least managed safely under their care. Therefore, not only should educators highlight important workflow updates to residents when they are out-rotated to tertiary hospitals, but there is also a need to train residents to treat patients as a whole so that residents can develop a rational approach for managing complex patients within their immediate context.

Secondly, there is an issue with confidentiality. Residents viewed confidentiality as a double-edged sword. While they understood the need to maintain patient confidentiality, they were torn by the extent of confidentiality should they need to discuss with other members of healthcare teams due to their lack of knowledge. This is a new area that has not been explored in preexisting research involving family medicine doctors. Perhaps this reflects the residents’ lack of experience dealing with such issues on confidentiality and the way to coordinate care within its boundaries.

In Singapore, family medicine residency is a three-year programme aimed at training doctors to become full-fledge family physicians who are proficient at coordinating care in the community. The findings from this study revealed that the challenges that our residents faced were similar to those faced by family physicians in other countries. These findings show that such challenges transcend geographical locations and all aspects of medical care.

While some of these challenges, such as time constraints, are systemic issues, other aspects, such as documentation and handovers, personal knowledge deficiencies, confidentiality and accessibility to partners, can be improved. This can be done through frequent updates on institutional workflows and community resources during residency meetings to residents. Residents could also run through mock case studies of complex patients to train themselves on areas of improvement in terms of handovers and documentation. Multidisciplinary team discussions with allied health professionals would also facilitate better understanding of the resources available and promote the exchange of ideas. Shared care plan discussion with patients’ involvement can provide them with an opportunity to express their healthcare decisions [23]. Furthermore, facilitating electronic integration and education of healthcare professionals to enhance integration of primary and secondary care of chronic diseases can facilitate better clinical outcomes^24^. Additionally, group discussions can be conducted to discuss commonly encountered issues of confidentiality to provide a better understanding among residents. Finally, common secured specialist-specific chat groups can be established to enhance accessibility when specialist inputs are needed.

It is with this hope that these timely interventions, when implemented in a longitudinal manner over the three years of residency, can culminate in the development of a family physician who is comfortable planning and coordinating care with relevant healthcare professionals within the community and tertiary setting.

### Strengths and limitations

To our knowledge, this is the first qualitative study on the challenges perceived by residents on care coordination in complex patients. The use of total population sampling with full participation of all the residents in the institution and in-depth interviews painted a complete picture by harnessing the views of all residents in the institution. This provided a bird’s eye view to their challenges in terms of coordinating care with these complex patients to inform future residency education interventions. The trustworthiness of the study was enhanced by striving for credibility, dependability, transferability and confirmability.

This study has a few limitations. This study looked at residents within an institution itself and not across the whole of Singapore; hence, it may not be transferable to the rest of the family medicine residents in other institutions. Additionally, the investigator who conducted the interviews analysed the transcripts, which may have been influenced by personal experiences with residency. To mitigate this bias, we employed reflexivity and had another two investigators (GYTS and LES) who was not involved in residency to analyse the data independently.

## Conclusion

The findings from this study identified both gaps in learning among residents and gaps in teaching residents with respect to understanding how to coordinate care, finding resources to better coordinate care and making sense of the boundaries of confidentiality where care coordination for complex patients is concerned. It is therefore vital to address these gaps early so that residents can be comfortable with collaborating with healthcare professionals from other institutions for their patients.

## Data Availability

The datasets used and analysed for this study are available from the corresponding authors upon reasonable request.

## Abbreviations

AIC: Agency of Integrated Care
WHO: World Health Organisation
NHGP: National Healthcare Group Polyclinics
ACGME: Accreditation Council for Graduate Medical Education
PDPA: Personal Data Protection Act
NEHR: National Electronic Health Records

## References

[1] Community Case Management Service (CCMS). The Agency for Integrated Care (AIC). (2019). https://fycs.org/wp-content/uploads/2023/04/CCMS-Referral-Form-V1.pdf Accessed 6 February 2021.

[2] Zulman D (2014). Quality of care for patients with multiple chronic conditions: the role of comorbidity interrelatedness. J Gen Intern Med.

[3] Vogeli C, Shields A (2007). Multiple chronic conditions: prevalence, health consequences, and implications for quality, care management, and costs. J Gen Intern Med.

[4] World Health Organisation. Continuity and coordination of care: a practice brief to support implementation of the WHO Framework on integrated people-centred health services. (2019). Accessed 4 March 2021.

[5] Stille C, Bell D (2015). Coordinating care across diseases, settings, and clinicians: a key role for the generalist in practice. Ann Intern Med.

[6] Friedman A, Shaw E (2016). Facilitators and barriers to care coordination in patient-centered Medical homes (PCMHs) from coordinators’ perspectives. J Am Board Fam Med.

[7] Ministry of Health, Singapore. Promoting healthier living while targeting specific subpopulations. (2022). https://www.moh.gov.sg/news-highlights/details/promoting-overall-healthier-living-while-targeting-specific-sub-populations. Accessed 15 May 2021.

[8] Adeniji C, Kenning C (2015). What are the core predictors of ‘hassles’ among patients with multimorbidity in primary care? A cross sectional study. BMC Health Serv Res.

[9] NHG Polyclinics. NHG Residency Family Medicine: Learning Experience. (2020). https://www.nhgeducation.nhg.com.sg/nhgresidency/programmes/training-and-residency-programmes/family-medicine/learning-experience. Accessed 8 June 2021.

[10] International ACGME. LLC. Family Medicine Milestones for Singapore. (2017) Accessed 20 February 2021.

[11] Øvretveit J. Does clinical coordination improve quality and save money? A summary of review and evidence. (2011) https://www.health.org.uk/sites/default/files/DoesClinicalCoordinationImproveQualityAndSaveMoneyVol1_summary.pdf Accessed 4 April 2021.

[12] Ministry of Health, Singapore. Updates on Phase 3 (Heightened Alert) Measures. (2021) https://www.moh.gov.sg/news-highlights/details/updates-on-phase-3-(heightened-alert)-measures. Accessed 8 September 2021.

[13] Bradshaw C, Doody O (2017). Employing a qualitative description Approach in Health Care Research. Glob Qual Nurs Res.

[14] Braun V (2006). Using thematic analysis in psychology. Qual Res Psychol.

[15] Tong A, Sainsbury P et al. Consolidated criteria for reporting qualitative research (COREQ): a 32–item checklist for interviews and focus groups. Int J Qual Health Care. 2007;19(6):349–57. College of Family Physicians, Singapore. Graduate Diploma in Family Medicine. (2023). https://www.cfps.org.sg/programmes/graduate-diploma-in-fm/. Accessed 18 September 2024. 10.1093/intqhc/mzm042 17872937

[16] Personal Data Protection Commission, Singapore. Advisory Guidelines for the Healthcare Sector. (2017). Accessed 6 October 2021.

[17] Kang S (2016). Spaced Repetition promotes efficient and effective learning: policy implications for instruction. Policy Insights Behav Brain Sci.

[18] Foo K, Sundram M (2020). Facilitators and barriers of managing patients with multiple chronic conditions in the community: a qualitative study. BMC Public Health.

[19] Hays R, Daker-White G (2017). Threats to patient safety in primary care reported by older people with multimorbidity: baseline findings from a longitudinal qualitative study and implications for intervention. BMC Health Serv Res.

[20] Dinsdale E, Hannigan A (2020). Communication between primary and secondary care: deficits and danger. Fam Pract.

[21] Sinnott C, McHugh S (2013). GPs’ perspectives on the management of patients with multimorbidity: systematic review and synthesis of qualitative research. BMJ Open.

[22] Bevilacqua G, Bolcato M. et.al. Shared care plan: an extraordinary tool for the personalisation of medicine and respect for self determination. Acta Biomed, 2021; 92(1). 10.23750/abm.v92i1.9597 PMC7975960 33682824

[23] Murtagh M, Mccombe G (2021). Integrating primary and secondary care to enhance chronic disease management: a scoping review. Int J Integr Care.

